# Anti-Synthetase Syndrome: A Case Report on the Elevated Risk of Pneumocystosis

**DOI:** 10.7759/cureus.63982

**Published:** 2024-07-06

**Authors:** Btissame Es-Sabbahi, Mounia Serraj, Mohammed Chakib Benjelloun, Mohamed ElBiaze, Bouchra Amara

**Affiliations:** 1 Pulmonology Department, Hassan II University Hospital, University of Sidi Mohammed Ben Abdellah, Fez, MAR

**Keywords:** corticosteroids, hiv-negative, pneumocystis jiroveci, pneumocystis jirovecii pneumonia, anti-synthetase syndrome

## Abstract

*Pneumocystis carinii* pneumonia (PCP), now referred to as *Pneumocystis jirovecii* pneumonia (PJP), occurs in immunocompromised patients. It is particularly associated with cellular immunodeficiency due to certain diseases or treatments. The risk of PCP is likely correlated with the severity of cellular immunity damage. However, excluding AIDS, the precise degree of immunosuppression required to develop PCP is not yet clearly understood.

We report the case of a 58-year-old patient who presented with progressively worsening dyspnea. The clinical examination revealed a SaO₂ of 88% on room air and the appearance of mechanic's hands. A thoracic CT scan showed interstitial lung disease (ILD). The immunological work-up was positive for antinuclear antibodies (ANA) and anti-JO-1 antibodies. Bronchoscopy with bronchoalveolar lavage (BAL) was performed, and the test for PJP came back positive.

## Introduction

Anti-synthetase syndrome is an autoimmune condition characterized by antibodies directed against aminoacyl-tRNA synthetase, along with clinical features that can include interstitial lung disease (ILD), myositis, Raynaud’s phenomenon, and arthritis [1]. The ILD in patients with anti-synthetase syndrome is often severe and rapidly progressive, determining the disease prognosis [2]. Patients with anti-synthetase syndrome may also be prone to serious infectious complications such as *Pneumocystis jirovecii* pneumonia (PJP), especially when undergoing immunosuppressive therapy. PJP occurs almost exclusively in immunocompromised patients and is an opportunistic infection that typically appears as a late complication in patients with HIV [3]. Since 1980, however, an increasing number of reports have described the occurrence of PCP in patients with connective tissue diseases (CTD) [4], with a higher incidence in myositis compared to other CTDs. However, the frequency of this complication remains unknown.

## Case presentation

We present a case of a 58-year-old patient, a bricklayer by profession, an occasional smoker, with a 17-year history of type 2 diabetes and a 15-year history of hypertension. For the past two years, he has been suffering from progressively worsening dyspnea, which becomes worse at rest, associated with a dry cough and diffuse joint pain. He consulted a physician who prescribed home oxygen therapy and occasional short courses of oral corticosteroids (less than three per year) during exacerbations of his symptoms.

After 18 months, he presented with worsening dyspnea, cough, sputum production, and weakness over the past four weeks. Clinical examination found tachypnea with an oxygen saturation of 88% in ambient air, improved to 95% on 2 liters of oxygen, and bilateral but asymmetrical crepitant rales on pulmonary auscultation. He also had Mechanic’s hands with a thickened, hyperkeratotic eruption along the fingers.

Arterial blood gas results on 2 liters of oxygen were as follows: pH 7.38; pCO2 47.5 mmHg; pO2 123 mmHg; Hematocrit (Hct) 43%; HCO3- 28 mmol/L; base excess (BE) 2.5 mmol/L; SaO2 94%.

Laboratory findings revealed hemoglobin of 14.4 g/dL (14-16 g/dl); hematocrit of 42.3% (40-50%); white blood cells 7800/μL (4000-10000/μL); neutrophils 4650/μL (2700-7000 /μL); lymphocytes 1970/μL (1500-4000μL); platelets 236,000/μL (150,000-450,000μL); C-reactive protein (CRP) 6 mg/L (< 5 mg/L); creatinine 8 mg/L (8-14 mg/L); urea 0.19 g/L (0.10-0.55 g/L); Pro-Brain Natriuretic Peptide (Pro-BNP) 55.38 pg/mL (<300 pg/mL); D-dimers 1030 ng/mL (< 500 ng/mL); HbA1C 7.3% (4-5.6%).

**Figure 1:**
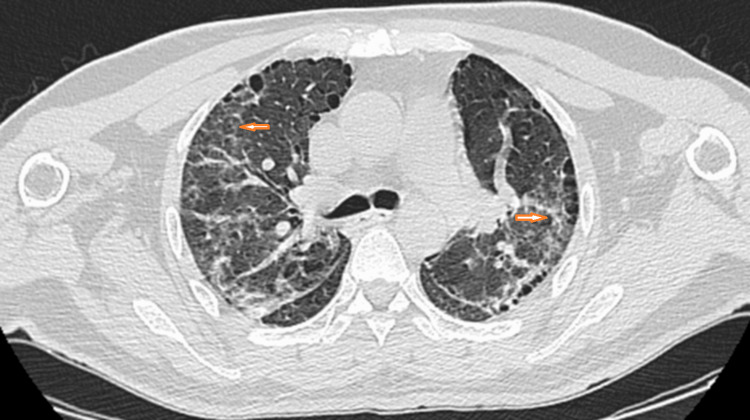
Axial CT scans of the chest with lung window settings, showing extensive ground-glass opacity.

**Figure 2:**
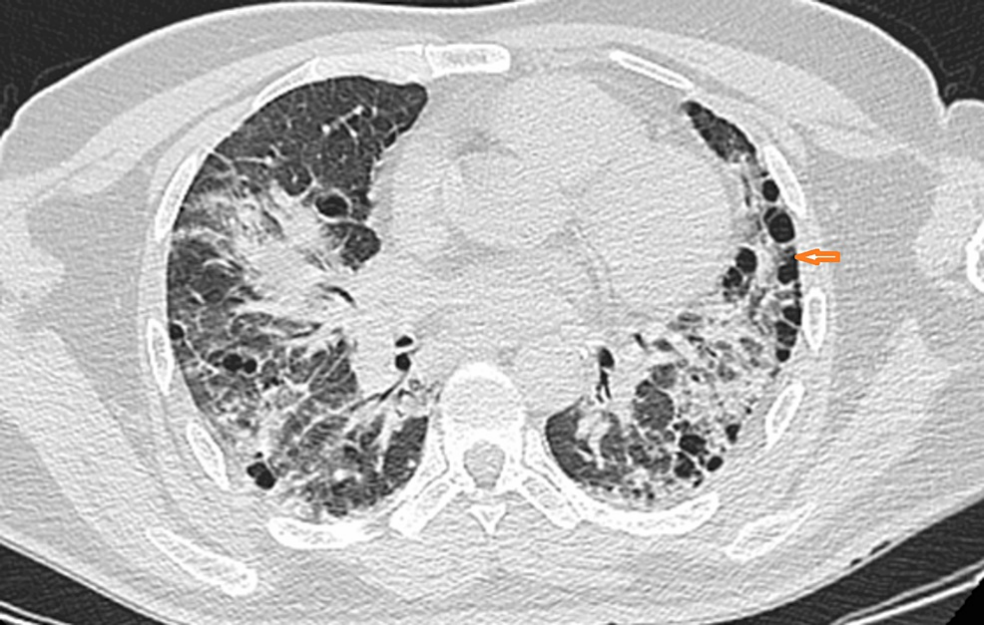
Axial CT scans of the chest with lung window settings showing honeycombing.

Thoracic CT angiography ruled out pulmonary embolism, while thoracic CT showed bilateral ground-glass opacities, cysts (Figure 1), and honeycombing without mediastinal lymphadenopathy (Figure 2). Antinuclear antibodies and anti-Jo-1 antibodies were positive, while capillaroscopy was normal.

Bronchoscopic examination revealed a normal appearance, direct microscopic examination revealed the presence of *Pneumocystis carinii*, and a bacterial study isolated *Haemophilus influenzae* (type b), also identified in the sputum, favoring bacterial pneumonia, especially in front of the condensation foci he had.

HIV serology was negative. Tumor markers were evaluated: B2-microglobulin 2.86 mg/L; lactate dehydrogenase (LDH) 492 IU/L; carcinoembryonic antigen (CEA) 2.68 ng/mL; cancer antigen 19-9 (CA19-9) 23.13 IU/mL; total prostate-specific antigen (PSA) 0.479 ng/mL. Cervico-thoraco-abdominal-pelvic CT did not reveal any suspicious lesions.

The diagnosis of antisynthetase syndrome was based on anti-Jo-1 positive antibodies, ILD, and mechanic’s hand.

The patient was treated with a curative dose of sulfamethoxazole-trimethoprim, and the negative result of the direct examination of induced sputum was confirmed after 10 days of treatment. Treatment continued for three weeks, then transitioned to a preventive dose. We opted for IV pulse steroid therapy; corticosteroid boluses were administered for three days, followed by oral administration at a dose of 1 mg/kg/day, along with IV cyclophosphamide injections every three weeks, as he had severe ILD with hypoxemia. The patient received six cyclophosphamide boluses with azathioprine relay, as well as gradual tapering of corticosteroids. Currently, he is on azathioprine 150 mg/day and cortisone 15 mg/day. Treatment progress has been favorable; currently, he saturates at 94% on room air.

## Discussion

In the 1980s, with the emergence of the HIV epidemic, the prevalence of pneumocystis surged, leading to its widespread recognition as an opportunistic infection causing severe pneumonia in immunocompromised individuals. At that point, antipneumocystis chemoprophylaxis became systematic in high-risk patients [3]. Even though there has been a decrease in occurrences among HIV-infected individuals over the past decade, pneumocystis pneumonia continues to be a significant concern for immunocompromised patients with various conditions requiring immunosuppression. This is attributed to the increased utilization of immunosuppressive medications in the treatment of autoimmune diseases, as well as post-transplantation, and in patients with hematological and solid malignancies [4]. However, data regarding the prevalence of PCP in connective tissue diseases other than Wegener’s granulomatosis are limited.

In 1994, Godeau B et al. conducted a retrospective analysis involving 10 adult medical units across five university hospitals in France over a decade. They investigated 34 cases of PCP in CTD patients, with the distribution as follows: Wegener’s granulomatosis (n=12), systemic lupus erythematosus (SLE) (n=6), polymyositis/dermatomyositis (n=5), polyarteritis nodosa (n=4), and others (n=7). Only two SLE patients who hadn't received cytotoxic drugs or corticosteroids were identified; the rest (32) were on corticosteroid treatment, with 24 also receiving cytotoxic drugs like cyclophosphamide and methotrexate [5]. Based on this study, PCP's estimated frequency was 16 cases per 10,000 patients per year in polyarteritis, 20 cases per 10,000 patients per year in inflammatory myopathy, 8 cases per 10,000 patients per year in SLE, and 1.3 cases per 10,000 patients per year in rheumatoid arthritis.

In 1998, Ward MM et al. utilized a state hospitalization registry in California from 1983 to 1994 to identify PCP patients with CTD, excluding those with AIDS. Among 240 patients enlisted, 223 were analyzed, with frequencies per 10,000 hospitalizations per year as follows: 89 in Wegener’s granulomatosis, 65 in polyarteritis nodosa, 27 in inflammatory myopathy, 12 in SLE, 8 in scleroderma, and 2 in rheumatoid arthritis [4].

Ishikawa Y et al. [6] performed an observational study in Japan, a country with a relatively high CTD-PCP incidence, focusing on pneumocystis cases in CTD patients. Among 67,890 admitted patients, 333 had CTD, predominantly primary vasculitis (n=116), inflammatory myositis (n=60), and SLE (n=49).

In 2014, Fillatre P et al. conducted a retrospective analysis of pneumocystosis cases admitted to Rennes University Hospital from 1990 to 2010, excluding HIV-positive patients [7]. They estimated incidence rates per 100,000 patient-years, revealing significant variations across different conditions. Notably, they emphasized high rates in polyarteritis nodosa (93.2) and granulomatosis with polyangiitis (71.9). Additionally, pneumocystosis incidence rates were elevated in patients with polymyositis/dermatomyositis (53.6 cases per 100,000 patients/year). In contrast, other inflammatory diseases exhibited lower incidence rates, with less than 25 cases per 100,000 patient-years for rheumatoid arthritis (<25), Sjögren's syndrome (<20), and sarcoidosis (<5).

These studies underscore a higher incidence of PCP in myositis compared to other CTDs such as sarcoidosis and rheumatoid arthritis. However, the influence of factors like corticosteroids and immunosuppressants requires further investigation. Notably, there is a lack of comparative studies assessing the impact of these risk factors, including corticosteroids and other immunosuppressants, on PCP incidence across different CTDs. Systematic chemoprophylaxis in these patients is likely to expose them to the drug's side effects. Indeed, a meta-analysis of patients receiving trimethoprim-sulfamethoxazole (TMP-SMX) prophylaxis revealed a significant incidence of adverse effects, highlighting the potential risks of widespread prophylactic treatment; the rate of discontinuation due to adverse events was 15.2%, with severe adverse events occurring in 3.1% of cases [8].

Corticosteroids and lymphopenia, associated with many CTDs, are the most common elements implicated as risk factors for the development of PJP. In the studies cited, most patients were on corticosteroids or other immunosuppressive therapy. However, in our case, the patient was not on long-term corticosteroids; he was only taking short courses of prednisone, which amounted to less than four courses per year of 40 mg/day for five days. Thus, most authors consider corticosteroids given at a daily dose of ≥20 mg of prednisone equivalent for at least four weeks as a risk factor for PJP [9]. On his CBC, there was no lymphopenia; the lymphocyte count was 1400 cells/µL. Therefore, there are likely other pathophysiological mechanisms beyond lymphopenia and therapy-induced immunosuppression that need to be investigated.

On the other hand, the challenge of diagnosing PCP in patients experiencing worsening respiratory conditions raises questions about the systematic screening for PCP in CTD, particularly in high-risk cases such as myositis.

## Conclusions

In conclusion, pneumocystis pneumonia remains a significant concern for immunocompromised patients, particularly those receiving corticosteroids and immunosuppressive therapy. While incidence rates vary across different connective tissue diseases, the lack of comparative studies underscores the need for further investigation into risk factors and preventative strategies. Collaborative efforts among healthcare professionals are essential for developing tailored approaches to PCP management and prevention in CTD patients.
